# Identification of a novel *Dlg2* isoform differentially expressed in IFNβ-producing plasmacytoid dendritic cells

**DOI:** 10.1186/s12864-018-4573-5

**Published:** 2018-03-12

**Authors:** Shafaqat Ali, Alexander Hoven, Regine J. Dress, Heiner Schaal, Judith Alferink, Stefanie Scheu

**Affiliations:** 1 Institute of Medical Microbiology and Hospital Hygiene, Heinrich, Heine University of Düsseldorf, Universitätsstraße 1, D-40225 Düsseldorf, Germany; 2 Cluster of Excellence EXC 1003, Cells in Motion, Waldeyerstraße 15, D-48149 Münster, Germany; 3 Singapore Immunology Network, Agency for Science, Technology, and Research (A*STAR), Singapore, 138648 Singapore; 4 Institute of Virology, Heinrich Heine University of Düsseldorf, Universitätsstraße 1, D-40225 Düsseldorf, Germany; 5 BMFZ (Biologisch-Medizinisches Forschungszentrum), Heinrich Heine University of Düsseldorf, Universitätsstraße 1, D-40225 Düsseldorf, Germany; 6 Department of Psychiatry, University of Münster, Albert-Schweitzer-Campus 1, D-48149 Münster, Germany

**Keywords:** *Dlg2*, PSD-93, IFNβ, Plasmacytoid dendritic cells, Isoforms

## Abstract

**Background:**

The murine discs large homolog 2 (DLG2; post synaptic density 93 (PSD-93); Chapsyn-110) is a member of the membrane-associated guanylate kinase (MAGUK) protein family involved in receptor assembly and associated with signaling enzymes on cell membranes. In neurons, DLG2 protein isoforms derived from alternatively spliced transcripts have been described to bind to NMDA (N-methyl-aspartate) receptors and K channels and to mediate clustering of these channels in the postsynaptic membrane. In myeloid cells of the immune system, such as dendritic cells (DCs), a lack of data exists on the expression or function of DLG2. In cDNA microarray transcriptome analyses, we found *Dlg2* highly expressed in a subpopulation of plasmacytoid DCs (pDCs) stimulated to produce type I interferons (IFNs) such as IFNβ.

**Results:**

Using RACE- and RT-PCR as well as immunoprecipitation followed by Western blotting we characterised the differential expression of the *Dlg2* splice variants in IFNβ-producing pDCs. Besides *Dlg2ɣ* this cell population expressed a novel short *Dlg2η* transcript we termed *Dlg2η3*. Our expression data were integrated into information from genome databases to obtain a novel and comprehensive overview of the mouse *Dlg2* gene architecture. To elucidate the intracellular localisation pattern of protein isoforms, ectopical expression analysis of fluorescently tagged DLG2 splice variants was performed. Here we found an enrichment of the larger isoform DLG2α1 at the plasma membrane while the newly identified shorter (DLG2η) isoform as well as DLG2ɣ were equally distributed throughout the cytoplasm. Additionally, DLG2η was also found in the nucleus. Analysis of *Dlg2*-knockout mice previously generated by deleting exon 9 surprisingly revealed that the protein for the novel DLG2η isoform was still expressed in the brain and in bone marrow-derived pDCs from mice carrying the homozygous deletion (*Dlg2*^*ΔE9/ΔE9*^).

**Conclusion:**

We describe a novel splice variant of the mouse *Dlg2* gene termed *Dlg2η* and define the differential expression pattern of DLG2 isoforms in IFNβ-producing pDCs. The presence of DLG2η protein in the CNS of *Dlg2*^*ΔE9/ΔE9*^ mice might influence the phenotype of these mice and has to be taken into account in the interpretation of results regarding the functional role of DLG2 in neuronal postsynaptic membranes.

**Electronic supplementary material:**

The online version of this article (10.1186/s12864-018-4573-5) contains supplementary material, which is available to authorized users.

## Background

Discs large homolog 2 (DLG2), also known as post synaptic density 93 (PSD-93) or Chapsyn-110, is a member of the membrane-associated guanylate kinase family of proteins (MAGUKs). Functionally, these proteins are best described as postsynaptic scaffold proteins binding neurotransmitter receptors and enzymes involved in the formation of signaling complexes in the postsynaptic density in neurons [1–5]. More specifically, MAGUKs control the trafficking of *N*-Methyl-D-aspartate (NMDA) and α-amino-3-hydroxy-5-methyl-4-isoxazole propionic acid (AMPA) type glutamate receptors to the synapse and thus excitatory synaptic transmission [2, 5–7]. In mammals five homologues of *Dlg2* are found - *Dlg1* (SAP-97, hDLG), *Dlg3* (SAP-102), *Dlg4* (PSD-95, SAP-90), *Dlg5,* and *Dlg6 (Mpp4)* [3–5, 7–11]. The respective contribution of the different *Dlg* homologues to synapse formation and function is still under debate.

Gene mutations in human *DLGs* have been shown to be causative for a spectrum of psychiatric disorders [3, 12]. Most prominently among these, mutations, copy number variations, or altered expression levels in four *DLG* homologues (*Dlg1*, *Dlg2*, *Dlg3* and *Dlg4*) have been implicated in the development of schizophrenia [13–19]. Further, *DLG3* mutations result in nonsyndromic X-linked mental retardation [20], while forms of autism are associated with mutations in Neuroligins, binding partners of *DLG4* [21]. Recently, *DLG2* has been associated with neurodevelopmental disorders in general and also with the pathogenesis of migraine [22, 23]. Important mechanistic insights have been gained by the analyses of mouse models with selective or combined deficiencies in *Dlg* homologues. Here, mutations in *Dlg2* and *Dlg4* lead to hypersocial behaviour and alterations in complex cognitive processes, while simple associative learning was impaired in *Dlg4*-deficient mice [8, 24].

All DLG homologues share a characteristic structure of protein-protein interaction domains. A variable N-terminal domain is followed by three PSD-95/discs large/zona occludens-1 (PDZ) domains and a src-homology 3 (SH3) domain that is linked to a guanylate kinase (GK) like domain [4, 5, 25, 26]. DLG6 here represents an exception in containing only one PDZ domain. PDZ domains typically bind to short amino acid motifs at the C-termini or internal β-finger motifs of interacting proteins such as receptors, ion channels, or enzymes [5, 27–29]. The SH3-GK domains are speculated to mediate oligomerization of DLGs [2, 5, 26].

Multiple isoforms exist for all six DLG homologues. Their transcripts are generated by using alternative transcription start sites and alternative splicing of exons. DLG isoforms exhibit differences in neuronal trafficking and fulfil specific roles in synaptic functioning [26, 30]. Of the six DLG homologues, DLG2 exhibits the highest number of variations in its N-terminal domains [25, 31]. The six N-termini of DLG2 so far described comprise two palmitoylated isoforms (α1 and α2), one L27 domain containing isoform (β), and three additional isoforms termed γ, δ, and ε [23, 25, 31].

In addition to the different N-termini, isoform variability of DLG2 in neurons is achieved by alternative splicing of exons coding for the linker region between the SH3 and the GK domain [25, 32]. This linker region might facilitate binding to different interaction partners depending on the splice variant expressed [26, 32]. Similarly, for DLG1, a specific interaction in this region with the actin/spectrin binding protein, protein 4.1, has been described that targets DLG1 to neuronal spines [33].

Knowledge of the expression pattern and function of splice variants of *Dlg2* outside the nervous system is very limited. Within the immune system only two broad scale transcription profiling approaches indicate significant expression levels of *Dlg2* in mast cells and splenic red pulp macrophages, respectively [34, 35]. So far, nothing is known about the expression or function of DLG2 in other cells of the immune system. In a transcription profiling approach we found *Dlg2* as highly expressed in pDCs that produce IFNβ [36]. The pDC subset of dendritic cells is considered to be specialized in the rapid production of high amounts of antiviral type I IFNs after activation but has a rather limited activity in antigen presentation and T cell priming as compared to conventional DCs [37, 38]. Recently we could show, using our IFNβ/YFP (*IFNβ*^*mob/mob*^) reporter mice, that only a small subset of these pDCs is responsible for the initial expression of type I IFNs after systemic activation of the pattern recognition receptor TLR9. The type I IFN-producing pDCs are equipped with a specific gene signature enabling them to control leukocyte recruitment and to coordinate early cellular immune responses [36]. Interestingly, *Dlg2* was found to be the transcript with the highest differential expression in these type I IFN-producing pDCs.

In this study, we present a revised annotation of the mouse genomic *Dlg2* locus and define a cell specific splicing pattern for *Dlg2* in murine pDCs. Additional to the previously described neuronal splice forms, we found a novel, shorter splice variant termed *Dlg2η3*. This isoform is specifically expressed in pDCs after stimulation with Toll like receptor 9 (TLR9) ligands. Further, we detected that protein expression of DLG2η was retained in the earlier published *Dlg2*^*ΔE9/ΔE9*^ mouse line [39] with unknown but possible functional implications for the phenotype of these mice.

## Methods

### Mice and in vivo treatments

*Dlg2*^*ΔE9/ΔE9*^ [39] mice were a kind gift of Seth G. N. Grant (Edinburgh University, Edinburgh, UK). Bicistronic Interferon β/YFP reporter knock-in mice (mob: messenger of IFN beta; *IFNβ*^*mob/mob*^) have been described previously [40]. *IFNβ*^*mob/mob*^, *Dlg2*^*ΔE9/ΔE9*^ mice, and their wild type (WT) littermates are on C57BL/6 N background and were kept under pathogen-free conditions. The mice were euthanized by cervical dislocation. The experiments were approved by the government of North-Rhine Westphalia. Where indicated, mice were injected i.v. with 10 μg CpG 1668 (TIB MOLBIOL) complexed to DOTAP (Roche) for 6 h, or as indicated.

### Generation and stimulation of bone marrow-derived pDCs

Bone marrow (BM)-derived FMS-like tyrosine kinase 3 ligand (Flt3L)-cultured pDCs were generated as previously described [40]. Cells were stimulated with 1 μM CpG 2216 (TIB MOLBIOL) complexed to DOTAP (Roche) for 6 h, or as indicated.

### Flow cytometry and cell sorting

Cells were analysed on a FACSCanto II (BD). DAPI was added for dead cell discrimination. FACS sorting was performed on a FACSAria (BD) after MACS (Miltenyi Biotec) depletion of CD3ε/CD19^+^ cells. Monoclonal antibodies against B220, CD3ε, CD11b, CD11c, CD19, CD27, CD86, and CD16/32 were purchased from BD, mPDCA-1 was purchased from BioLegend.

### Gene expression analyses

RNA was isolated from FACS-sorted in vivo differentiated or BM-derived Flt3L cultured pDCs using the NucleoSpin RNA Isolation Kit (MACHEREY-NAGEL). Expression of *Dlg2* and *Ifnb* was analysed in IFNβ/YFP^+^ and IFNβ/YFP^—^ pDCs using quantitative RT-PCR (qRT-PCR). qRT-PCR was performed using the TaqMan Master Kit with the Universal Probe Library Set (Roche) or the MESA GREEN qPCR MasterMix Plus (Eurogentec) on an iQ5 or CFX96 (Bio-Rad). The primer sequences used for qRT-PCR are as follows: mDlg2-fwd AAACGCTCCCTGTATGTCAGA, mDlg2-rev CCCCATCTAGTGTGACCCTTC, Ifnb-fwd CAGGCAACCTTTAAGCATCAG, Ifnb-rev CCTTTGACCTTTCAAATGCAG, β-actin-fwd TGACAGGATGCAGAAGGA, and β-actin-rev CGCTCAGGAGCAATG.

### Mammalian cell culture and transient transfection

NIH3T3 cells were purchased from ATCC. The HEK293 derived cell line 293FT was purchased from Thermo Scientfic. Both cell lines were maintained in Dulbecco’s modified Eagle’s medium (DMEM, Gibco) containing 10% heat inactivated fetal bovine serum (FBS; Pan Biotech) and 2 mM L-glutamine (Biochrom) at 10% CO_2_. Cells were transiently transfected with plasmids as indicated in figure legends by a slightly modified polyethylenimine (PEI; Aldrich) transfection method [41].

### Immunoprecipitation and western blotting

First, BM-derived Flt3L cultured pDCs were generated from *Dlg2*^*ΔE9/ΔE9*^ mice or WT littermates as described above. After 7 days of differentiation in Flt3L containing medium, 12 × 10^7^ BM-derived Flt3L cultured DCs were either left untreated or stimulated with CpG 2216 complexed to DOTAP for 16 h. At the end of the stimulation time cells were washed and lysed with RIPA lysis buffer for 30 min at 4 °C. Insoluble fractions were removed before the supernatant was incubated with anti-DLG2 (Abcam) antibody coupled to protein G agarose beads (Sigma-Aldrich) for 16 h at 4 °C with gentle rotation. Washed beads were resuspended in Laemmli sample buffer and heated to 95 °C for 10 min. In parallel, transiently transfected HEK293 cells were washed with PBS and lysed as described above. After SDS/PAGE, DLG2 was detected in a Western blot with mouse anti-DLG2 (Millipore) or rabbit anti-DLG2 (Abcam) antibodies.

### Expression plasmids

*pDlg2α-EGFP, pDlg2γ-EGFP and pDlg2η-EGFP* encode the murine DLG2α, DLG2γ, and DLG2η isoforms, respectively, C-terminally fused to EGFP. The respective transcription unit in the *pEGFP-N1* vector (Clontech) is under control of the CMV promoter. *Dlg2 α, γ,* and *η* isoforms were amplified by PCR using Dlg2αM1s_KEco (TCGGAATTCGCCACCATGTTCTTTGCATGTTATTGTGC), Dlg2γM1s_KEco (AGAGAATTCGCCACCATGTTACCGACTTTCGATATGC), Dlg2ηM1s_KEco (TGAGAATTCGCCACCATGATGAACCACAGCATGAGC) as sense primer respectively and Dlg2-stopas_Bam (TGCGGATCCAACTTCTCCTTTGAGGGAATC) as antisense primer. Amplified PCR products were ligated into the *pEGFP-N1* vector (Clontech) at the *Eco*RI and *Bam*HI sites. The *pIFNβ-Luc* reporter plasmid encodes the firefly luciferase under the control of the *Ifnb* minimal promoter and was used for the *Ifnb* reporter gene assays. *pRL-CMV* (Promega) encodes renila luciferase under the control of CMV promoter. *pRL-CMV* was used as an internal transfection control in reporter gene assays. *pFlag-IRF7* encodes an N-terminally Flag tagged interferon regulatory factor 7 (IRF7). *phTBK1-Flag-His* encodes a C-terminally Flag and His tagged human TANK binding kinase 1 (TBK1). *pCI-TPI-WT-4H* encodes human triosephosphate isomerase 1 (TPI1) under CMV promoter [42]. Primers and cloning details are available on request.

### Reporter gene assay

For IRF7-dependent reporter gene assays, 1.25 × 10^5^ NIH3T3 cells were cotransfected with 50 ng *pIFNβ-Luc*, 5 ng *pRL-CMV*, 10 ng *pFlag-IRF7*, 40 ng *phTBK1-Flag-His* and 0, 70, 140, or 280 ng *Dlg2* isoforms or *TPI1* encoding plasmids in 24-well plates. The total amount of plasmid DNA was always maintained by adding empty vector. 20 h after transfection, cells were lysed with 100 μl passive lysis buffer (Promega). Luciferase activity was measured using the dual luciferase assay system (Promega) as described earlier [43].

### Immunostaining and microscopy

BM-derived Flt3L cultured pDCs were either kept untreated or stimulated with 1 μM CpG 2216 for 24 h. Cells were transferred on coverslips by centrifugation. NIH3T3 cells were cultured and transfected on cover slips. Paraformaldehyde fixed cells were stained for different markers as shown in the figures. DAPI was used as nuclear marker. Abs against Na/K ATPase and GFP/YFP were purchased from Abcam, CD16/32, DLG2 and mPDCA-1 were purchased from BD, Millipore, and Miltenyi Biotec, respectively. Images were taken on a LSM 780 (Zeiss) confocal microscope and processed using Adobe Photoshop and ZEN 2011/12 software.

### RACE PCR and isotype specific PCR

BM-derived Flt3L cultured pDCs were generated from *IFNβ*^*mob/mob*^ mice. Cells were stimulated with 1 μM CpG 2216 for 6 h and IFNβ/YFP^+^ and IFNβ/YFP^—^ pDCs were FACS sorted. Rapid amplification of cDNA ends (RACE) was performed using SMARTer® RACE 5′/3′ kit (Takara Clontech) and *mDlg2* 5RACE GSP1 (GATTACGCCAAGCTTCCTCTGTTTCCTTCATGGCTTCAC) or *mDlg2* 5RACE GSP2 (GATTACGCCAAGCTTTGTCGTTGTCAGAGGTGCAGTAGC) primers. Amplified PCR products were cloned using In-Fusion HD cloning kit (Takara Clontech). Primers used for isotype specific RT-PCR analysis of *Dlg2* isoforms are given in Table 1.

**Table 1:** Primer used for isotype specific RT-PCR

Name	Exon	Sequences (5′-3′)
Dlg2α1 fwd	α1	CTGAGCTCTCACTCAGTGCCTTC
Dlg2α2 fwd	α2	AGCTGCCGCTCTGTCTAGGCTG
Dlg2δ fwd	δ	GGGAGGAAGCCTTTCTATGCAG
Dlg2rev	11	CGGTGGCCCATAAGGATCAGT
Dlg2α0/β fwd	4	AAGGCAAATGCCCAGCCCAG
Dlg2α0/β rev	9	TAGAGCCGGCTTCCTTGAG
Dlg2γ fwd	γ	GTGAAGAAGCTATGCAACACGCGT
Dlg2γ rev	14	CGAGTTGCAGTACTGTGCTGG
Dlg2ε fwd	ε	GCCAACTGGATGTGTGTGAGCCG
Dlg2ε rev	9	CACAACAGTCTCCAATATGGGTCGC
Dlg2ζ1 fwd	ζ1	AGCTGGCTGTGTTTCCAGTGCC
Dlg2ζ2 fwd	ζ2	TCCCCAGAGCCCATATAGAGCCA
Dlg2η1 fwd	η1	ACACAACAGCCCCCAAACCAC
Dlg2η2 fwd	η2	ACCGGACAGGAGACAGGAGA
Dlg2η rev	17	TGACATACAGGGAGCGTTTCT
Dlg2 E18 fwd	18	AGAAGGGTCACACTAGATGG
Dlg2 E19 fwd	19	GTGGAAAGAAAGGAGCGTG
Dlg2 E20 fwd	20	CCATTCTACAAGAACAAGGAGC
Dlg2 E21 fwd	21	ATTAGGTGACGACGGTTATGG
Dlg2 E20 rev	20	ATCACTGGTTTCCTGCTCAC
Dlg2 E21 rev	21	GAGTCTTTGTTCCATAACCGTC
Dlg2 E22 rev	22	AGCTACTTTCGCTATCGCTG
Dlg2 E23 rev	23	GCCTCGTGACAGGTTCATAG

### Statistical analysis

Data are shown as individual values with mean ± SEM. Differences between two groups were tested using t-test. A *p*-value < 0.05 was considered as significant. Multiple comparisons were performed with One-way ANOVA followed Bonferroni’s Multiple comparison Test or Two-way ANOVA followed by Bonferroni posttests. GraphPad Prism 5.0 was used to perform statistical analyses and to prepare graphs.

## Results

### Correlation of DLG2 and IFNβ expression in mouse pDCs

**Fig. 1:**
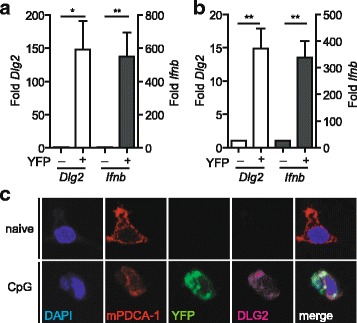
High expression of DLG2 in IFNβ-producing pDCs. (**a**) Analysis of *Dlg2* expression in splenic pDCs. IFNβ/YFP^+^ and IFNβ/YFP^—^ splenic pDCs were ex vivo FACS sorted from *IFNβ*^*mob/mob*^ mice 6 h after i.v. injection with CpG. *Dlg2* (white bars) and *Ifnb* (black bars) mRNA expression was analysed by quantitative RT-PCR. (**b**) *Dlg2* expression in in vitro BM-derived pDCs. BM-derived DCs were generated from *IFNβ*^mob/mob^ mice in Flt3L cultures. IFNβ/YFP^+^ and IFNβ/YFP^—^ BM-derived pDCs were FACS sorted 6 h after stimulation with CpG. *Dlg2* (white bars) and *Ifnb* (black bars) mRNA expression was analysed by quantitative RT-PCR. Data shown in (**a**) and (**b**) is fold mRNA quantity in IFNβ-producing pDCs relative to IFNβ non-producers. Data shown is mean ± standard error of mean (SEM) from four independent experiments (each sample is pooled from 12 (**a**) or 6 (**b**) mice. Statistical differences between IFNβ/YFP^+^ and IFNβ/YFP^—^ were analysed by two tailed, unpaired t test. ns: *P* > 0.05, *: *P* < 0.05, **: *P* < 0.01, ***: *P* < 0.001. (**c**) Immunofluorescence images of DLG2 expression in BM-derived Flt3L cultured pDCs left untreated (naïve; upper panel) and 24 h after CpG (CpG; lower panel) stimulation. Cells were stained with antibodies against DLG2 and mPDCA-1 and a YFP-crossreacting antibody against GFP. Data shown is one representative experiment out of three independent experiments with comparable results

In our recent microarray based transcriptome analyses *Dlg2* was found to be differentially expressed in IFNβ-producing pDCs [36]. To verify our previously generated microarray data by an independent method we first performed quantitative analysis of mouse *Dlg2* mRNA expression on ex vivo FACS sorted splenic or in vitro Flt3L cultured BM-derived pDCs (Fig. 1a, b). To induce and to track IFNβ expression in pDCs, IFNβ/YFP reporter mice (*IFNβ*^*mob/mob*^) were injected i.v. with the TLR9 ligand CpG and IFNβ-expressing YFP^+^ and IFNβ-non-producing YFP^—^ splenic pDCs were FACS sorted 6 h after stimulation. Quantitative RT-PCR analysis showed a higher expression of *Ifnb* in IFNβ/YFP^+^ vs. IFNβ/YFP^—^ pDCs (549.7 ± 144.8 fold) confirming the purity of the sorted cells as well as the functionality of the IFNβ/YFP reporter model. For *Dlg2*, markedly higher mRNA levels (148.0 ± 42.6 fold) were measured in IFNβ/YFP^+^ vs. IFNβ/YFP^—^ ex vivo sorted pDCs corroborating our earlier transcriptome data (Fig. 1a). Next, we tested whether *Dlg2* is also highly expressed in type I IFN-producing pDCs independent of the anatomical splenic environment in vitro. For this, BM-derived Flt3L cultured pDCs were stimulated with CpG for 6 h to activate type I IFN production and FACS sorted for IFNβ/YFP expression. In this in vitro system, as well, elevated *Dlg2* mRNA levels (14.8 ± 3.0 fold) correlated well with a higher expression of *Ifnb* (337 ± 63.0 fold) in IFNβ/YFP^+^ as compared to IFNβ/YFP^—^ pDCs (Fig. 1b). Of note *Dlg2* expression was also detectable in conventional DCs (cDCs) albeit to a lesser extent. Also, in cDCs CpG stimulation did not lead to an increase in *Dlg2* mRNA levels (Additional file 1).

Similar to pDCs, B cells constitutively express TLR9 [44]. TLR9 stimulation with CpG results in increased CD27 surface expression in a sub-population of B cells which correlates with higher antibody-secretion in these cells [45]. We asked whether in B cells higher CD27 surface levels correlate also with increased *Dlg2* expression after CpG stimulation. Therefore, we analyzed the expression of *Dlg2* in CD27-positive versus CD27-negative B cell sub-populations after TLR9 stimulation. *Dlg2* expression, however, did not correlate with CD27 expression in these cells and was around 150 fold less as compared to BM-derived IFNβ/YFP-producing pDCs (Additional file 2). Of note, *Ifnb* mRNA levels in both B cell subpopulations were also 250 fold lower as compared to the professional type I IFN producers. These data suggest a pDC-specific elevated expression of *Dlg2* upon TLR9 activation.

Finally, coexpression of DLG2 and IFNβ was analysed at protein level in BM-derived Flt3L cultured pDCs left untreated or stimulated for 24 h with CpG. Here, immunofluorescent staining for DLG2 was detected exclusively in CpG stimulated pDCs that exhibited a positive signal for IFNβ/YFP (Fig. 1c). These data indicate that *Dlg2* mRNA as well as DLG2 protein is highly differentially expressed in IFNβ-expressing pDCs as compared to pDCs not producing IFNβ ex vivo as well as in vitro.

### *Dlg2* splice variants expressed in IFNβ-expressing pDCs

**Fig. 2:**
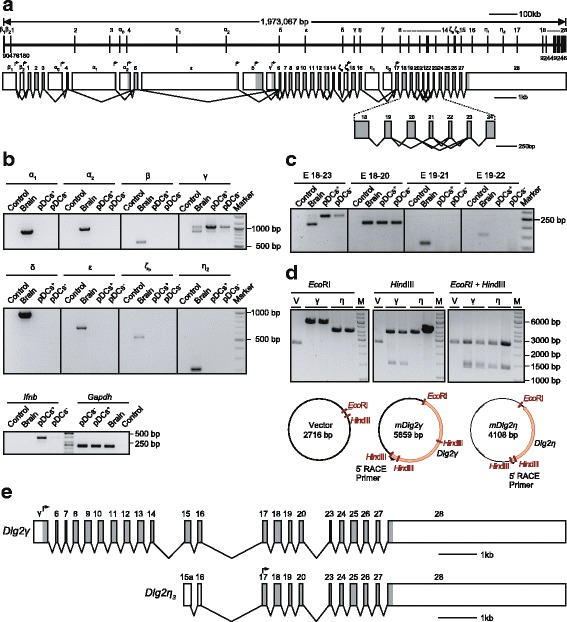
Analysis of *Dlg2* isoforms expressed in IFNβ/YFP^+^-producing pDCs. (**a**) Gene architecture and alternatively spliced transcripts of the *Dlg2* gene. (**a**, upper graph) Genomic position of *Dlg2* gene is presented. Exons are shown as vertical bars and introns as thin horizontal lines. Introns and exons are drawn to scale. Smaller exons (less than 1 point line space) are not to scale. (**a**, lower graph) Exons are shown as boxes and are drawn to scale. Exons are named or numbered as indicated. Alternative exon-exon-junctions are indicated with connecting lines. Grey boxes show protein coding regions whereas empty boxes represent untranslated mRNA regions. (**b**) RT-PCR of *Dlg2* N-terminal isoforms in IFNβ/YFP^+^ and IFNβ/YFP^—^ BM-derived pDCs (upper and middle panel). Flt3L cultures from six pooled *IFNβ*^*mob/mob*^ mice were stimulated with CpG for 6 h and FACS-sorted for YFP^+^ (pDC^+^) and YFP^—^ pDCs (pDC^—^). Naïve brain from C57BL/6 N mice was used as positive and not reversely transcribed RNA from YFP^+^ pDCs as negative controls (Control). Lower panel shows *Gapdh* and *Ifnb* expression in the respective cDNA samples indicating successful stimulation and sorting of pDCs as well as equal template amounts. (**c**) SH3-GUK linker isoforms of *Dlg2* in RT-PCR. SH3-GUK region was amplified using cDNA samples as described above in (**b**). (**d**) Restriction analysis of *Dlg2* clones generated after 5´-RACE PCR. Empty vector or selected 5´-RACE clones were digested either with *Eco*RI (left), *Hin*dIII (middle), or with both restriction enzymes (right). Lower panel shows the plasmid maps for the clones shown in the upper panel. (**e**) Exon-intron structure of the *Dlg2* isoforms expressed in pDCs. Exons are shown as boxes and are drawn to scale as shown in A (lower part)

Six N-terminal protein variants have been described for mouse DLG2, including two palmitoylated isoforms and an L27 domain containing isoform [25, 31]. Transcripts initiated at alternative promoters are responsible for the expression of these different N-terminal domains. Additionally, alternative splicing leads to variations in the usage of exons coding for the SH3-GK linker region of DLG2 [25]. Here, we generated an updated version of the mouse *Dlg2* gene architecture by integrating current information available from the ENSEMBL and NCBI genome databases (Fig. 2a and Additional file 3). The terms for the alternative N-terminal protein domains α, β, ε, δ, and γ were adopted as suggested by Krüger et al. [25]. In contrast to the previously published gene structure [25], we found two additional exons coding for distinct N-terminal L27 containing β-isoforms. We termed these exons, at the very 5′ end of *Dlg2,* β1 and β2. The β1 exon is spliced onto exon 1 or alternatively to the β2 exon which then is spliced onto exon 1 (Additional file 3). The first possibility results in a protein translated from the start codon in the β1 exon. The second splice variant generates a premature stop codon for translation events initiated from the start codon in the β1 exon. Thus, the DLG2β2 protein is produced using the start codon in the β2 exon (Fig. 2a). In a sequence available in the database under the annotation XM_006507768 both these exons, β1 and β2, are combined into a larger single exon retaining the otherwise intronic sequence. From this sequence, also only the DLG2β2 isoform can be translated. Exons 1 to 3 are shared by all L27 containing β-isoforms. For the palmitoylated α-isoforms we found three exons (α0, α1, and α2) coding for distinct N-termini (Fig. 2a and Additional file 3). This is in contrast to previous analyses where only two α exons had been described [25]. The here defined exon α0 is spliced onto exon 4 that is shared by all β isoforms whereas both, exon α1 and α2, are spliced directly onto exon 5 which is common for all α- and β-isoforms. Exon 5 as well as the unique exons coding for the N-termini of the isoforms ε and δ can each be spliced onto exon 6. In contrast, the exon coding for the N-terminus of the isoform γ is spliced alternatively onto exon 6 or directly onto exon 7. Further analyses of recent database entries revealed the existence of two additional N-terminal domains we termed ζ and η (Fig. 2a and Additional file 3). The two consecutive exons ζa, which is non-coding, and ζb, which contains the translational start codon for the N-terminus of the ζ-isoform, are spliced onto exon 15. This exon is shared also by the α-, β-, ε-, δ-, and γ-isoforms. Finally, the open reading frame of the η-isoform is preceded by either one of the two non-coding exons η1 or η2 which are spliced onto exon 17, the most 5′ exon present in all known isoforms of *Dlg2*. The variable linker sequences between the SH3 and the GK domain are encoded by the alternatively spliced exons 20 to 23 according to our updated gene annotation.

We next defined the expression of the different N-terminal isoforms in pDCs. For this we performed RT-PCR analyses on sorted Flt3L cultured BM-derived IFNβ/YFP^+^ or IFNβ/YFP^—^ pDCs 6 h after stimulation with CpG (Fig. 2b). RNA isolated from whole brains of C57BL/6 N mice served as control. While transcripts for all α-, β-, ε-, δ-, γ-, ζ-, and η1/2-isoforms could be amplified from brain, in pDCs only the γ-transcripts were detectable. Corroborating our results from quantitative RT-PCR analyses using primers that detect all known isoforms (Fig. 1b), also higher expression levels were found using primers specific for the γ-isoform in IFNβ-producing as compared to non-producing pDCs. In brain, two alternatively spliced variants for *Dlg2γ*, which are derived from alternative inclusion of exon 6, could be amplified at comparable levels. In pDCs, inclusion of exon 6 appeared relatively more frequent. We went on to define alternative exon usage within the SH3/GK-linker region using primers specific for exons 18 through 23 (Fig. 2c). In RNA preparations from the whole brain, in accordance with the literature [25], alternatively spliced transcripts containing exons 20 through 23 could be detected. We failed, however, to detect transcripts containing either exon 21 or 22 in pDCs. From this RT-PCR analyses we concluded that only the γ-isoform lacking exons 21 and 22 is expressed in pDCs. These findings emphasized further a pDC specific splicing pattern for *Dlg2*. Of note, similar expression pattern could be detected in IFNβ-producing cDCs (Additional file 4 and data not shown) although expression levels of *Dlg2* were around 25-fold less in cDCs as compared to pDCs (Additional file 1). To be also able to identify so far unknown 5′ ends of *Dlg2* splice variants in pDCs we performed 5′ RACE PCR using a 3′-primer binding within exon 28 on RNA isolated from IFNβ-producing Flt3L cultured BM-derived pDCs (Fig. 2d). We recovered several clones containing cDNAs for the γ-isoform but no clones containing the α-, β-, ε-, δ-, or ζ-isoforms. Interestingly, the majority of the 5′-RACE clones, however, harboured cDNAs from a novel *Dlg2* isoform that uses a 5′ transcription start site 28 nucleotides upstream of exon 15. This novel exon was termed exon 15a. The translational start codon for this isoform is localised in exon 17 and identical to the translational start codon of the η-isoform identified by our in silico analyses. We termed this novel *Dlg2* isoform *Dlg2η3* (Fig. 2e). Taken together, *Dlg2* expression in pDCs is restricted to a known γ- and a novel η3-isform (MF276899, and MF276900) with skipping of exons 21 and 22. Since these exons, when included, code for the SH3/GK-linker region (Fig. 2) the pDC specific DLG protein isoforms contain a shorter linker region that might mediate specific protein-protein interactions.

### Protein expression of DLG2 isoforms in murine pDCs

**Fig. 3:**
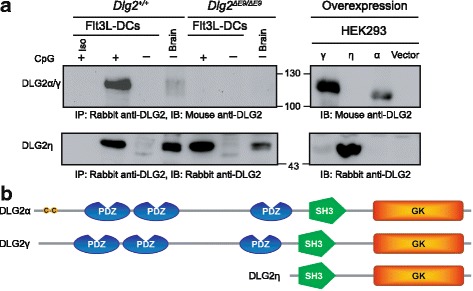
Protein expression of a novel η-isoform of DLG2 in pDCs. (**a**) Detection of DLG2 isoforms at protein level in pDCs. DLG2 was immunoprecipitated from BM-derived Flt3L cultured DCs and brain lysates (left panel) from WT or *Dlg2*^*ΔE9/ΔE9*^ mice. The cells were left untreated or stimulated with CpG for 6 h. Immunoprecipitated DLG2 isoforms were analysed by DLG2 specific immunoblotting (upper panel); Iso: Isotype control antibody. In parallel, recombinant murine DLG2, isoforms (indicated) were expressed in HEK293 cells after transient transfection (right panel). DLG2 isoforms were analysed by immunoblotting as described above. (**b**) Protein domain organization of the DLG2 α-, γ-, and η-isoform expressed in pDCs. Indicated are the double cysteine-motif of the palmitoylation site within the α-isoform, and the PSD-95/Dlg/ZO-1 (PDZ), Src-homology-3 (SH3), and Guanylate Kinase (GK) domains. Protein length and relative positions of the domains are drawn to scale

Following the analysis of the *Dlg2* splicing pattern and the definition of *Dlg2γ* and *Dlg2η3* transcript isoforms in type I IFN-producing pDCs, we further elucidated DLG2 expression at the protein level. Therefore, we performed immunoprecipitation followed by Western blotting for different DLG2 isoforms on pDCs and brain tissue from wildtype (WT) and *Dlg2* deficient mice generated before [39] (Fig. 3a, left). Due to the lack of isoform specific antibodies overexpression of recombinant γ-, η-, and α-isoforms served as size references (Fig. 3a, right). In protein lysates from BM-derived Flt3L cultured cells from WT mice stimulated for 16 h with CpG a band of approximately 120 kDa was detectable corresponding well with the band size of the recombinantly expressed γ-isoform. In brain lysates, at lower levels, expression of at least 2 bands of approximately 110 and 120 kDa, the same size of the γ- and α-isoforms from recombinantly expressed controls, could be found. Both of these bands were not detectable in protein lysates from *Dlg2* knock out (*Dlg2*^*ΔE9/ΔE9*^) mice generated by deleting exon 9. Of note, in brain and stimulated Flt3L cultures from WT as well and *Dlg2*^*ΔE9/ΔE9*^ mice a band of approximately 50 kDa was detectable. This band corresponded in size with the novel η-isoform expressed in transfected HEK293 cells. Thus, the supposed knock out for *Dlg2* harbouring a deletion for a region within the second PDZ domain [39] retains expression of the short η-isoform of DLG2 lacking all three PDZ domains in brain and Flt3L cultured pDCs (Fig. 3b).

### Intracellular localisation of DLG2 α-, γ-, and η-isoforms

**Fig. 4:**
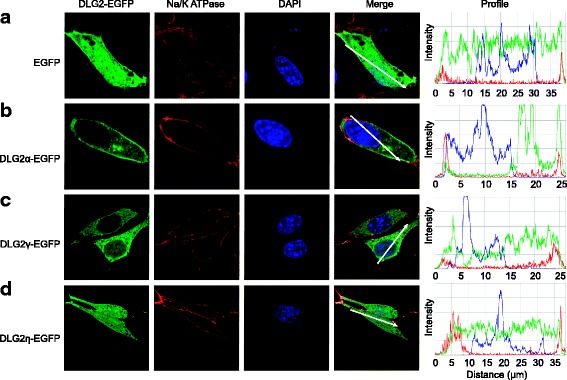
Localisation of murine DLG2 isoforms ectopically expressed in NIH3T3 cells. EGFP (**a**) or murine DLG2 isoforms DLG2α (**b**), DLG2γ (**c**) or DLG2η (**d**) fused to EGFP were expressed in NIH3T3 cells. A day after transient transfection the cells were fixed and stained for Na/K ATPase as a cell membrane marker and nuclei (DAPI). Stained cells were analysed by confocal microscopy. Single channel views (left and middle panels) or merged views are displayed. Signal intensities of different markers are shown (right panel; profile) for the area under the arrow (merge) as analysed by ZEN software. Data shown is one representative picture out of a series of pictures analysed in at least three interdependent experiments

The different DLG2 isoforms and the DLG homo- and orthologues of the MAGUK family have been described to localise differentially to specific intracellular compartments [33, 46]. To elucidate the preferential localisation of the pDC-specific γ- and η-isoforms we generated expression plasmids encoding GFP-fusion proteins of these DLG2 isoforms. Additionally, the brain-specific α-isoform was cloned as a GFP-fusion protein as reference. NIH3T3 cells were transiently transfected with these constructs (Fig. 4). Overexpression of GFP alone resulted in an equal distribution of the fluorescence throughout the cytoplasm and the nucleus (Fig. 4a). The C-terminally GFP-tagged α-isoform preferentially localised to the cytoplasmic membrane as identified by costaining with the Na/K-ATPase serving as a membrane marker (Fig. 4b). The α-isoform of DLG2 includes an N-terminal palmitoylation site that enables association with membrane lipids. Thus, the cytoplasmic membrane-associated localisation observed here proves the functionality of this experimental system. Additionally, the α-isoform was enriched within an intracytoplasmatic compartment likely representing the Golgi apparatus due to its perinuclear localisation. In contrast, the γ- and η-isoforms showed a distinct localisation when expressed as GFP-fusion proteins. While the γ-isoform of DLG2 preferentially localised within the cytoplasm and to a lesser extent to the nucleus (Fig. 4c) the η-isoform was found evenly distributed between the cytoplasmic and nuclear compartments (Fig. 4d). This specific subcellular localisation hints at distinct cellular functions of the respective DLG2 isoforms.

**Fig. 5:**
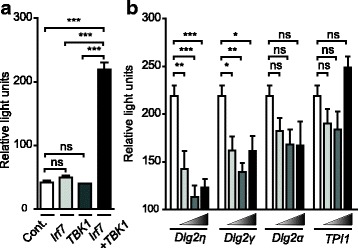
The effect of ectopic expression of *Dlg2* isoforms on *Ifnb* expression. (**a**). Effect of IRF7 activation on *Ifnb* promoter activity. IRF7 or TBK1 were expressed alone or in combination in the presence of *Ifnb* reporter plasmid. Control (Cont.) shows the basal activity of *Ifnb* promoter in NIH3T3 cells. (**b**). Effect of expression of *Dlg2* isoforms on *Ifnb* promoter activity. Increasing amounts of *Dlg2* isoforms or *TPI* gene were transiently transfected along with *Irf7*, *Tbk1* encoding plasmids and an *Ifnb* reporter vector in NIH3T3 cells. *Ifnb* promoter activity was measured from cell lysates one day after transfection. Data shown are ratios of relative light unites of Firefly luciferase (under the control of the *Ifnb* promoter) / Renila luciferase (under the control of the CMV promoter) from one representative experiment performed in quadruplicates out of three independent experiments. Data shown are the means ±SEM. Significance was analyzed by either One-way ANOVA followed by Bonferroni’s Multiple Comparison Test (**a**) or Two-way ANOVA followed by Bonferroni posttests (**b**). ns: *P* > 0.05, *: *P* < 0.05, **: *P* < 0.01, ***: *P* < 0.001

Since increased expression of DLG2 was observed in IFNβ-expressing pDCs and significant levels of DLG2η localized to the nucleus, it can be hypothesized that DLG2 isoforms directly or indirectly regulate the expression of IFNβ upon CpG stimulation. To test this and due to the lack of availability of cells deficient in the DLG2η isoform, we analyzed the effects of ectopic expression of *Dlg2* isoforms on *Ifnb* promoter activity in a luciferase reporter gene assay. After CpG stimulation TLR9 signaling leads to a TANK binding kinase 1 (TBK1) induced interferon regulatory factor 7 (IRF7) activation which is a prerequisite for type I interferon expression in pDCs. Overexpression of neither IRF7 nor TBK1 alone were able to significantly activate the *Ifnb* promoter in NIH3T3 cells. However, co-expression of both molecules increased the *Ifnb* promoter activity (Fig. 5a) indicating a pDC like TLR9 signal transduction for *Ifnb* promoter activity in NIH3T3 cells. Using these conditions, we additionally overexpressed different *Dlg2* isoforms in increasing concentrations. Expression of the *Dlg2η* isoform and to lesser extent *Dlg2γ* dampen the *Ifnb* promoter activity whereas overexpression of *Dlg2α* and *TPI1* used as an unrelated control did not affect the *Ifnb* promoter activity (Fig. 5b). These results are an initial hint that DLG2η and DLG2γ isoforms might function as negative regulators of *Ifnb* expression.

## Discussion

In a recent transcriptome analysis we found *Dlg2* as highly differently expressed in pDCs that produced the antiviral cytokines of the type I IFN family after activation of the pattern recognition receptor TLR9 [36]. We verified the increased expression of *Dlg2* in in vitro BM-derived as well as ex vivo isolated mouse pDCs after stimulation with the TLR9 ligand CpG. The expression detected on mRNA as well as on protein level correlated with the production of type I IFN in these pDCs. In other cell types tested, such as cDCs and B cells, no increase in *Dlg2* levels was observed. This points towards a pDC-specific *Dlg2* upregulation after TLR9 stimulation. So far, expression of DLG2 has not been described in this myeloid cell type of the immune system. Rather, as a MAGUK family member, DLG2 is best characterized as a scaffolding protein in the post-synaptic density of neurons. There, DLGs are known to interact with cell adhesion molecules such as N-cadherins that have important roles in structural and functional aspects of synapses [5, 47]. In immune cells also, cell adhesion proteins have vital functions controlling cell migration and homing. It can be envisaged that DLG2 might be involved in pDC migration and localization within lymphatic as well as peripheral tissues.

Outside the nervous system, DLG2 expression is less well characterized. In expression profiling screens of diverse mouse cell lines and primary immune cell types [34, 35] *Dlg2* was detected in splenic red pulp macrophages [48] and more prominently in mast cells [49]. However, activated pDCs were not included in these expression profiling screens. Mast cells are known to be critically involved in the initiation and promotion of airway inflammation and asthma. Interestingly, in a recent genome wide analysis for gene-environment interaction effects on childhood asthma differential methylation and expression of *DLG2* was found associated with air pollution exposure [50]. In light of this, further expression and functional analyses of DLG2 in mast cells could reveal important new implications of this MAGUK protein in type 2 driven immunopathologies.

DLG2 has further been implicated in immunopathological mechanisms in a focal ischemic stroke mouse model by exacerbating the cerebral ischemic injury and the induction of pro-inflammatory cytokines and downregulation of anti-inflammatory mediators [51, 52]. Here reduced levels of TNF and IL-6 but increased amounts of the anti-inflammatory IL-10 were found in the brains of *Dlg2* deficient as compared to WT mice after ischemia/reperfusion injury. In this setting iNOS and COX-2 were even downregulated after ischemia induction in the absence of DLG2 while both factors were found increased in the ischemic brains of WT animals [52]. This study linked the known DLG2-mediated neuronal excitotoxicity [53] with the release of iNOS, TNF, and IL-6 presumably by microglia. The exact causal interactions of neurons and microglia and the role of DLG2 therein remain to be elucidated. Based on our findings of DLG2 expression in pDCs, microglia or other brain infiltrating myeloid cells have to be interrogated for a possible direct involvement of DLG2 in pro- and anti-inflammatory cytokine production.

DLG2 can be expressed in multiple isoforms with variable N-terminal domains and alternatively spliced internal exons coding for the SH3-GK linker region [25, 26]. Six N-terminal variants of DLG2 are expressed, albeit at varying levels, in different brain regions [25]. In contrast, our data show that pDCs only express two N-terminal variants, DLG2γ and DLG2η. While the shorter DLG2η isoform contains only an SH3 and the GK domain, in the DLG2γ isoform three additional PDZ domains are present at the N-terminus. The DLG2γ isoform neither contains a palmitoylation nor a L27 domain found in the α- and β-isoforms, respectively. PDZ domains bind specific C-terminal sequences in target proteins [2, 5]. For the PDZ domains 1 and 2 of DLG2 inwardly rectifying K^+^ channels and the GluN2B subunit of NMDA receptors have been identified as interaction partners in neurons. The third PDZ domain binds Neuroligins and the Src tyrosin kinase Fyn that regulates synaptic functions of NMDA receptors [4, 54]. The SH3 domains are commonly known as protein-protein interaction modules in a variety of proteins with widely divergent functions ranging from cell differentiation in embryonic development to cell signalling in immune cells [55]. Finally, the GK domain of MAGUK proteins is catalytically inactive and rather binds phosphorylated proteins as interaction partners, while the proposed orthologue in yeast, the *Saccharomyces cerevisiae* guanylate kinase (Guk1) catalyses the phosphorylation of GMP to GDP using ATP as the phosphate donor [2, 5, 56]. The SH3-GK linker region is suggested to mediate oligomerization within the MAGUK protein family [2, 5, 26, 57]. A future task will be the identification of specific binding partners of DLG2γ and DLG2η in pDCs. A first promising candidate might be Fyn as in pDCs recently this kinase together with another Src family kinase Lyn have been shown to be essential in constitutive and TLR-mediated signaling events [58]. For the DLG2 homologue DLG4 an interaction with nNOS is described. In neurons, it targets this enzyme and thereby formation of NO from L-arginine selectively to the NMDA receptor [2]. It is tempting to speculate that DLG2 in pDCs might be involved in the generation of NO thereby exhibiting direct immune effector functions.

The specific subcellular localization of alternatively spliced variants of DLG2 may lead to insights towards their respective functions. As a scaffold protein, it might direct signal transduction complexes to different intracellular sites thereby modulating the outcome of signalling cascades. Indeed, we found a preferential localization for DLG2γ within the cytoplasm while DLG2η was detectable also in the nuclear compartment. Interestingly, the MAGUK family protein CASK has been described to be able to translocate into the nucleus and there to regulate neuronal gene expression [59]. While our initial promoter activation experiments suggest a negative regulatory impact of DLG2 on IFNβ expression after TLR9 activation and its involvement in a negative feedback loop, the precise functions for DLG2η in transcriptional regulation in pDCs and its mechanisms remain to be elucidated.

During our DLG2 expression analyses we detected protein expression of the novel DLG2η isoform in the brain of *Dlg2*^*ΔE9/ΔE9*^ mice widely used for the analysis of DLG2-dependent biological processes [39]. As whole brain lysates were analysed for residual DLG2 expression in *Dlg2*^*ΔE9/ΔE9*^ mice we could not differentiate between neurons, glia, and other cell types of the CNS. It remains to be elucidated if the DLG2η detected is expressed specifically by neurons or other cells of the brain of *Dlg2*^*ΔE9/ΔE9*^ mice. Nevertheless, these observations have to be taken into account in the interpretation of unexpected findings in the *Dlg2*^*ΔE9/ΔE9*^ mouse line. For example, although DLG2 is thought to be the only DLG expressed in cerebellar Purkinje neurons, in *Dlg2*^*ΔE9/ΔE9*^ mice no defects in the synaptogenesis were observed [39]. Based on these findings it was assumed that the DLG homologues are not essential for synapse development in specific brain regions. Data from our studies indicate that in *Dlg2*^*ΔE9/ΔE9*^ mice expression of the η-isoform is retained in the brain. While the lack of PDZ domains makes it rather unlikely, it cannot be excluded that the short η-isoform is able to fulfil essential functions in synapse formation. Novel insights regarding the functions of DLG2 in cerebellar Purkinje neurons and also the redundancy of DLG2 and DLG4 in cognitive processes might be gained from complete *Dlg2* knock outs to be generated in the future.

## Conclusions

Taken together, here we describe for the first time expression of DLG2 in pDCs that represent the major cellular source of type I interferons in antiviral immune responses. We define the differential expression pattern of a novel splice variant, *Dlg2η,* in these cells and integrate this information into an updated annotated exon structure for the mouse *Dlg2*. Analysis of CNS tissue revealed presence of DLG2η protein in previously generated *Dlg2*^*ΔE9/ΔE9*^ mice. A yet to be established potential role of this isoform in pDCs and other myeloid cells as well as neurons awaits further clarification.

## Additional files

Additional file 1: Expression of *Dlg2* in IFNβ/YFP-producing pDCs and cDCs. (PDF 428 kb)

Additional file 2: Expression of *Dlg2* in B cells. (PDF 446 kb)

Additional file 3: Proposed *Dlg2* exon names/numbers, length, and location within the genomic sequence and accession numbers of reference sequences they are contained in. (DOCX 44 kb)

Additional file 4: *Dlg2* isoforms expressed in IFNβ-producing pDCs and cDCs. (PDF 560 kb)

## Abbreviations

ATP: Adenosine triphosphate
BM: Bone marrow
CASK: Calcium/calmodulin dependent serine protein kinase
CNS: Central nervous system
COX-2: Cyclooxygenase-2
CpG: 5**′**-cytosine-phosphate-guanine-3′
DCs: Dendritic cells
DLG: Discs large homolog
DOTAP: N-[1-(2,3-Dioleoyloxy)propyl]-N,N,N-trimethylammonium methyl-sulfate
EGFP: Enhanced green fluorescent protein
Flt3L: FMS-like tyrosine kinase 3 ligand
GDP: Guanosine diphosphate
GK: Guanylate kinase
GMP: Guanosine monophosphate
Guk1: Guanylate kinase 1
IFN: Interferon
IL: Interleukin
iNOS: Inducible nitric oxide synthase
IRF: Interferon regulatory factor
L27: Lin2 Lin7 domain
MAGUK: Membrane-associated guanylate kinase
MOB: Messenger of interferon beta
NMDA: N-methyl-D-aspartate
nNOS: Neuronal nitric oxide synthase
NO: Nitric oxide
PCR: Polymerase chain reaction
pDCs: Plasmacytoid dendritic cells
PDZ: PSD-95/discs large/zona occludens-1 domains
RACE: Rapid amplification of cDNA ends
SH3: Src-homology 3
TBK1: TANK binding kinase 1
TLR: Toll like receptor
TNF: Tumour necrosis factor
WT: Wildtype
YFP: Yellow fluorescent protein
