# Supplementary material for: Identification of a novel Dlg2 isoform differentially expressed in IFNβ-producing plasmacytoid dendritic cells
Source: BMC Genomics. 2018 Mar 12;19:194. doi: 10.1186/s12864-018-4573-5 (PMC6389146; doi:10.1186/s12864-018-4573-5)
Supplement: Supplementary file 1 — Expression of Dlg2 in IFNβ/YFP-producing pDCs and cDCs. (PDF 428 kb) [file 12864_2018_4573_MOESM1_ESM.pdf]

Additional file 1

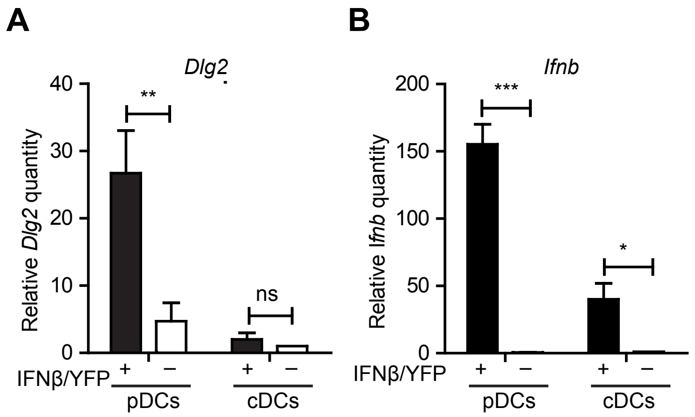

Additional file 1. Expression of *Dlg2* in IFNβ/YFP-producing pDCs and cDCs. BM-derived pDCs and cDCs were differentiated in Flt3L cultures. BM-derived cells were left untreated or stimulated with 1μM CpG 2216 ODN complexed to DOTAP and separated into IFNβ/YFP-positive vs. IFNβ/YFP-negative cells by FACS sorting. Expression of *Dlg2* (A) and *Ifnb* (B) were analyzed by qPCR. Data shown are relative quantities ($2^{-\Delta\Delta Ct}$) combined from three independent experiments. Black bars indicate the expression of *Dlg2* (A) or *Ifnb* (B) in the IFNβ/YFP-producing cells whereas white bars represent the expression of *Dlg2* (A) or *Ifnb* (B) in the IFNβ/YFP-non-producing cells. Data shown are mean values ± SEM. Differences between groups were tested using Two-way ANOVA followed by Bonferroni posttests. ns: P> 0.05, *: P<0.05, **: P<0.01, ***: P<0.001.
