# Supplementary material for: Identification of a novel Dlg2 isoform differentially expressed in IFNβ-producing plasmacytoid dendritic cells
Source: BMC Genomics. 2018 Mar 12;19:194. doi: 10.1186/s12864-018-4573-5 (PMC6389146; doi:10.1186/s12864-018-4573-5)
Supplement: Supplementary file 2 — Expression of Dlg2 in B cells. (PDF 446 kb) [file 12864_2018_4573_MOESM2_ESM.pdf]

Additional file 2

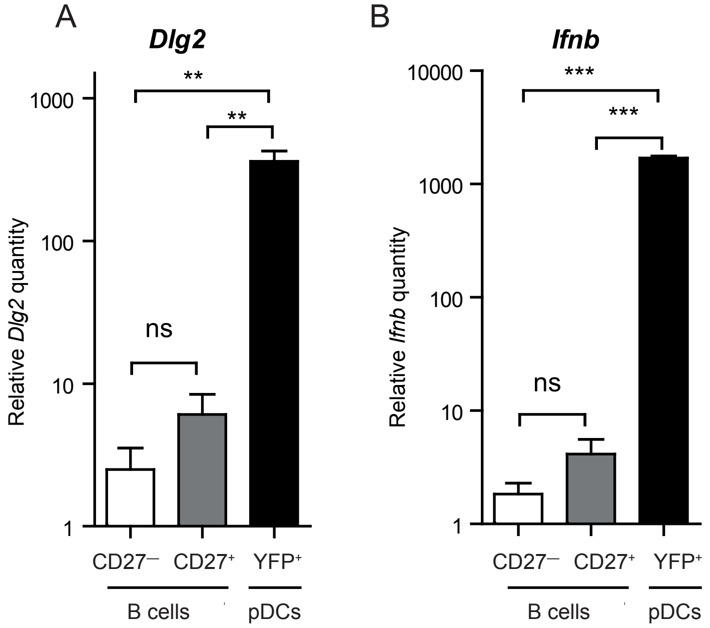

Additional file 2. Expression of *Dlg2* in B cells. Murine splenic cells were *ex vivo* stimulated with CpG (1µM) complexed to DOTAP. $CD27^{+}$ and $CD27^{-}$ B cells ($CD3^{-}CD11c^{-}CD19^{+}B220^{+}$) were FACS sorted. *Dlg2* and *Ifnb* expression was analyzed by qPCR. FACS sorted IFNβ/YFP^{+} cells were used as control. Data shown are relative quantities ($2^{-\Delta\Delta Ct}$) from one experiment performed in three biological replicates. Data shown are mean values ±SEM. Differences between groups were tested using One-way ANOVA followed Bonferroni's Multiple Comparison Test. A P value <0.05 was considered significant. ns: P>0.05, *: P<0.05, **: P<0.01, ***: P<0.001
