# Supplementary material for: Identification of a novel Dlg2 isoform differentially expressed in IFNβ-producing plasmacytoid dendritic cells
Source: BMC Genomics. 2018 Mar 12;19:194. doi: 10.1186/s12864-018-4573-5 (PMC6389146; doi:10.1186/s12864-018-4573-5)
Supplement: Supplementary file 3 — Proposed Dlg2 exon names/numbers, length, and location within the genomic sequence and accession numbers of reference sequences they are contained in. (DOCX 44 kb) [file 12864_2018_4573_MOESM3_ESM.docx]

| **Additional file 3**  **Proposed *Dlg2* exon names/numbers, length, and location within the genomic sequence and accession numbers of reference sequences they are contained in** | | | | | |
| --- | --- | --- | --- | --- | --- |
| **Exon** | **Location in genomic sequence** | **Length (bp)** | **Accession number for mRNA reference sequence** | **Accession number for coded protein reference sequence** | **Functional domains and Features** |
| β_1_ | 7:90476180-90476811 | 632 | XM_006507776 | XP_006507839 | Start,  L27 |
|  | 7:90476183-90476811 | 629 | XM_006507777 | XP_006507840 | Start,  L27 |
|  | 7:90476185-90476811 | 627 | XM_006507769, XM_006507767, XM_006507771, XM_006507770 | Non coding for XM_006507769,  XP_006507830, XP_006507834, XP_006507833 | Start,  L27 |
|  | 7:90476187-90476811 | 625 | XM_006507772 | XP_006507835 | Start,  L27 |
|  | 7:90476189-90476811 | 623 | XM_006507773 | XP_006507836 | Start,  L27 |
| β_2_ | 7:90476185-90477875 | 1691 | XM_006507768 | XP_006507831 | Start,  L27 |
|  | 7:90477718-90477875 | 156 | XM_006507769 | XP_006507832 | Start,  L27 |
| 1 | 7:90504806-90504937 | 132 | XM_006507776, XM_006507777, XM_006507768, XM_006507769, XM_006507767, XM_006507771, XM_006507770, XM_006507772, XM_006507773 | XP_006507839, XP_006507840, XP_006507831, XP_006507832, XP_006507830, XP_006507834, XP_006507833, XP_006507835, XP_006507836 | L27 |
| 2 | 7:90731895-90732040 | 146 | XM_006507776, XM_006507777, XM_006507768, XM_006507769, XM_006507767, XM_006507771, XM_006507770 XM_006507772, XM_006507773 | XP_006507839, XP_006507840, XP_006507831, XP_006507832, XP_006507830, XP_006507834, XP_006507833, XP_006507835, XP_006507836 | L27 |
| 3 | 7:90852668-90852763 | 96 | XM_006507776, XM_006507777, XM_006507768, XM_006507769, XM_006507767, XM_006507771, XM_006507770, XM_006507772, XM_006507773 | XP_006507839, XP_006507840, XP_006507831, XP_006507832, XP_006507830, XP_006507834, XP_006507833, XP_006507835, XP_006507836 |  |
| α_0_ | 7:90887055-90887719 | 665 | XM_006507779 | XP_006507842 | Start,  Predicted palmitoylation at C3 and C5 |
| 4 | 7:90915460-90915534 | 75 | XM_006507776, XM_006507777, XM_006507768, XM_006507769, XM_006507767, XM_006507771, XM_006507770, XM_006507772, XM_006507773, XM_006507779 | XP_006507839, XP_006507840, XP_006507831, XP_006507832, XP_006507830, XP_006507834, XP_006507833, XP_006507835, XP_006507836, XP_006507842 |  |
| α_1_ | 7:91088999-91091048 | 2050 | XM_006507784 | XP_006507847 | Start,  Predicted palmitoylation at C5 and C7 |
|  | 7:91089000-91091048 | 2049 | XM_006507783 | XP_006507846 | Start,  Predicted palmitoylation at C5 and C7 |
|  | 7:91090726-91091048 | 323 | ENSMUST00000146563 |  | Start,  Predicted palmitoylation at C5 and C7 |
|  | 7:91090728-91091048 | 321 | ENSMUST00000074273 | ENSMUSP00000073885, E9Q2L2 | Start,  Predicted palmitoylation at C5 and C7 |
|  | 7:91090786-91091048 | 263 | NM_011807, ENSMUST00000107196 | NP_035937, ENSMUSP00000102814, Q91XM9 | Start,  Predicted palmitoylation at C5 and C7 |
| α_2_ | 7:91262487-91262995 | 509 | XM_006507781, XM_017322227, XM_006507782 | XP_006507844, XP_017177716, XP_006507845 | Start,  Palmitoylation at C10 and C16 |
| 5 | 7:91449754-91449915 | 162 | NM_011807, XM_006507776, XM_006507777, XM_006507768, XM_006507769, XM_006507767, XM_006507771 XM_006507772, XM_006507773, XM_006507779, XM_006507783, XM_006507781, XM_017322227, ENSMUST00000107196, ENSMUST00000074273, ENSMUST00000146563 | NP_035937, XP_006507839, XP_006507840, XP_006507831, XP_006507832, XP_006507830, XP_006507834, XP_006507835, XP_006507836, XP_006507842, XP_006507846, XP_006507844, XP_017177716, ENSMUSP00000102814, ENSMUSP00000073885, Q91XM9, E9Q2L2 |  |
|  | 7:91449757-91449915 | 159 | XM_006507770, XM_006507784, XM_006507782 | XP_006507833, XP_006507847, XP_006507845 |  |
| ε | 7:91538625-91543191 | 4567 | XM_006507786 | XP_006507849 | Start, |
| δ | 7:91672025-91672954 | 930 | XM_006507778, XM_006507780 | XP_006507841, XP_006507843 | Start,  Predicted palmitoylation at C35 and C92 but with low score. |
|  | 7:91672492-91672954 | 463 | ENSMUST00000152139= AK046525 |  | Start |
| γ | 7:91711420-91711798 | 379 | ENSMUST00000107193 | ENSMUSP00000102811, D3YUZ8 | Start |
|  | 7:91711425-91711798 | 374 | XM_006507785, MF276899 | XP_006507848 | Start |
|  | 7:91711447-91711798 | 352 | XM_011241786 | XP_011240088 | Start |
| 6 | 7:91733091-91733144 | 54 | NM_011807, XM_006507776, XM_006507777, XM_006507768, XM_006507769, XM_006507767, XM_006507771, XM_006507770 XM_006507772, XM_006507773, XM_006507779, XM_006507784, XM_006507783, XM_006507781, XM_017322227, XM_006507782, XM_006507786, XM_006507778, XM_006507780, XM_006507785, ENSMUST00000107196, ENSMUST00000074273, ENSMUST00000146563, ENSMUST00000152139, MF276899 | NP_035937, XP_006507839, XP_006507840, XP_006507831, XP_006507832, XP_006507830, XP_006507834, XP_006507833, XP_006507835, XP_006507836, XP_006507842, XP_006507847, XP_006507846, XP_006507844, XP_017177716, XP_006507845, XP_006507849, XP_006507841, XP_006507843, XP_006507848, ENSMUSP00000102814, ENSMUSP00000073885, Q91XM9, E9Q2L2 |  |
| 7 | 7:91810479-91810529 | 51 | NM_011807, XM_006507776, XM_006507777, XM_006507768, XM_006507769, XM_006507767, XM_006507771, XM_006507770, XM_006507772, XM_006507773, XM_006507779, XM_006507784, XM_006507783, XM_006507781, XM_017322227, XM_006507782, XM_006507786, XM_006507778, XM_006507780, XM_006507785, XM_011241786, ENSMUST00000107196, ENSMUST00000074273, ENSMUST00000107193, ENSMUST00000146563, ENSMUST00000152139, MF276899 | NP_035937, XP_006507839, XP_006507840, XP_006507831, XP_006507832, XP_006507830, XP_006507834, XP_006507833, XP_006507835, XP_006507836, XP_006507842, XP_006507847, XP_006507846, XP_006507844, XP_017177716, XP_006507845, XP_006507849, XP_006507841, XP_006507843, XP_006507848, XP_011240088, ENSMUSP00000102814, ENSMUSP00000073885, ENSMUSP00000102811, Q91XM9, E9Q2L2, D3YUZ8 | PDZ1 |
| 8 | 7:91872376-91872500 | 125 | NM_011807, XM_006507776, XM_006507777, XM_006507768, XM_006507769, XM_006507767, XM_006507771, XM_006507770, XM_006507772, XM_006507773, XM_006507779, XM_006507784, XM_006507783, XM_006507781, XM_017322227, XM_006507782, XM_006507786, XM_006507778, XM_006507780, XM_006507785, XM_011241786, ENSMUST00000107196, ENSMUST00000074273, ENSMUST00000107193, ENSMUST00000146563, ENSMUST00000152139, MF276899 | NP_035937, XP_006507839, XP_006507840, XP_006507831, XP_006507832, XP_006507830, XP_006507834, XP_006507833, XP_006507835, XP_006507836, XP_006507842, XP_006507847, XP_006507846, XP_006507844, XP_017177716, XP_006507845, XP_006507849, XP_006507841, XP_006507843, XP_006507848, XP_011240088, ENSMUSP00000102814, ENSMUSP00000073885, ENSMUSP00000102811, D3YUZ8, Q91XM9, E9Q2L2 | PDZ1 |
| 9 | 7:91900735-91900904 | 170 | NM_011807, XM_006507776, XM_006507777, XM_006507768, XM_006507769, XM_006507767, XM_006507771, XM_006507770, XM_006507772, XM_006507773, XM_006507779, XM_006507784, XM_006507783, XM_006507781, XM_017322227, XM_006507782, XM_006507786, XM_006507778, XM_006507780, XM_006507785, XM_011241786, ENSMUST00000107196, ENSMUST00000074273, ENSMUST00000107193, ENSMUST00000146563, ENSMUST00000152139, MF276899 | NP_035937, XP_006507839, XP_006507840, XP_006507831, XP_006507832, XP_006507830, XP_006507834, XP_006507833, XP_006507835, XP_006507836, XP_006507842, XP_006507847, XP_006507846, XP_006507844, XP_017177716, XP_006507845, XP_006507849, XP_006507841, XP_006507843, XP_006507848, XP_011240088, ENSMUSP00000102814, ENSMUSP00000073885, ENSMUSP00000102811, Q91XM9, E9Q2L2, D3YUZ8 | PDZ1  PDZ2 |
| 10 | 7:91939999-91940135 | 137 | NM_011807, XM_006507776, XM_006507777, XM_006507768, XM_006507769, XM_006507767, XM_006507771, XM_006507770 XM_006507772, XM_006507773, XM_006507779, XM_006507784, XM_006507783, XM_006507781, XM_017322227, XM_006507782, XM_006507786, XM_006507778, XM_006507780, XM_006507785, XM_011241786, ENSMUST00000107196, ENSMUST00000074273, ENSMUST00000107193, ENSMUST00000146563, ENSMUST00000152139, MF276899 | NP_035937, XP_006507839, XP_006507840, XP_006507831, XP_006507832, XP_006507830, XP_006507834, XP_006507833, XP_006507835, XP_006507836, XP_006507842, XP_006507847, XP_006507846, XP_006507844, XP_017177716, XP_006507845, XP_006507849, XP_006507841, XP_006507843, XP_006507848, XP_011240088, ENSMUSP00000102814, ENSMUSP00000073885, ENSMUSP00000102811, Q91XM9, E9Q2L2, D3YUZ8 | PDZ2 |
| 11 | 7:91965596-91965740 | 145 | NM_011807, XM_006507776, XM_006507777, XM_006507768, XM_006507769, XM_006507767, XM_006507771, XM_006507770, XM_006507772, XM_006507773, XM_006507779, XM_006507784, XM_006507783, XM_006507781, XM_017322227, XM_006507782, XM_006507786, XM_006507778, XM_006507780, XM_006507785, XM_011241786, ENSMUST00000107196, ENSMUST00000074273, ENSMUST00000107193, ENSMUST00000146563, ENSMUST00000152139, MF276899 | NP_035937, XP_006507839, XP_006507840, XP_006507831, XP_006507832, XP_006507830, XP_006507834, XP_006507833, XP_006507835, XP_006507836, XP_006507842, XP_006507847, XP_006507846, XP_006507844, XP_017177716, XP_006507845, XP_006507849, XP_006507841, XP_006507843, XP_006507848, XP_011240088, ENSMUSP00000102814, ENSMUSP00000073885, ENSMUSP00000102811, Q91XM9, E9Q2L2, D3YUZ8 | PDZ2 |
| 12 | 7:91968118-91968256 | 139 | NM_011807, XM_006507776, XM_006507777, XM_006507768, XM_006507769, XM_006507767, XM_006507771, XM_006507770, XM_006507772, XM_006507773, XM_006507779, XM_006507784, XM_006507783, XM_006507781, XM_017322227, XM_006507782, XM_006507786, XM_006507778, XM_006507780, XM_006507785, XM_011241786, ENSMUST00000107196, ENSMUST00000074273, ENSMUST00000107193, ENSMUST00000146563, ENSMUST00000152139, MF276899 | NP_035937, XP_006507839, XP_006507840, XP_006507831, XP_006507832, XP_006507830, XP_006507834, XP_006507833, XP_006507835, XP_006507836, XP_006507842, XP_006507847, XP_006507846, XP_006507844, XP_017177716, XP_006507845, XP_006507849, XP_006507841, XP_006507843, XP_006507848, XP_011240088, ENSMUSP00000102814, ENSMUSP00000073885, ENSMUSP00000102811, Q91XM9, E9Q2L2, D3YUZ8 |  |
| 13 | 7:91997166-91997321 | 156 | NM_011807, XM_006507768, XM_006507769, XM_006507767, XM_006507771, XM_006507770, XM_006507772, XM_006507773, XM_006507779, XM_006507784, XM_006507783, XM_006507781, XM_017322227, XM_006507782, XM_006507786, XM_006507778, XM_006507780, XM_006507785, ENSMUST00000107196, ENSMUST00000074273, ENSMUST00000146563, MF276899 | NP_035937, XP_006507831, XP_006507832, XP_006507830, XP_006507834, XP_006507833, XP_006507835, XP_006507836, XP_006507842, XP_006507847, XP_006507846, XP_006507844, XP_017177716, XP_006507845, XP_006507849, XP_006507841, XP_006507843, XP_006507848, ENSMUSP00000102814, ENSMUSP00000073885, Q91XM9, E9Q2L2 |  |
|  | 7:91997166-91998815 | 1650 | ENSMUST00000152139  Retained intron |  | stop |
| 14 | 7:92040798-92040866 | 69 | NM_011807, XM_006507776, XM_006507777, XM_006507768, XM_006507769, XM_006507767, XM_006507771, XM_006507770, XM_006507772, XM_006507773, XM_006507779, XM_006507784, XM_006507783, XM_006507781, XM_017322227, XM_006507782, XM_006507786, XM_006507778, XM_006507780, XM_006507785, XM_011241786, ENSMUST00000107196, ENSMUST00000074273, ENSMUST00000107193, ENSMUST00000146563, MF276899 | NP_035937, XP_006507839, XP_006507840, XP_006507831, XP_006507832, XP_006507830, XP_006507834, XP_006507833, XP_006507835, XP_006507836, XP_006507842, XP_006507847, XP_006507846, XP_006507844, XP_017177716, XP_006507845, XP_006507849, XP_006507841, XP_006507843, XP_006507848, XP_011240088, ENSMUSP00000102814, ENSMUSP00000073885, ENSMUSP00000102811, Q91XM9, E9Q2L2, D3YUZ8 |  |
| ζ_a_ | 7:92062394-92062459 | 66 | NM_001243046, ENSMUST00000098308 | Non coding |  |
| ζ_b_ | 7:92066257-92066406 | 150 | NM_001243046, ENSMUST00000098308 | NP_001229975, ENSMUSP00000095910, D3YWU0 | Start |
| 15a | 7:92088534-92088718 | 185 | MF276900 | Non coding |  |
| 15 | 7:92088562-92088718 | 157 | NM_011807, NM_001243046, XM_006507776, XM_006507777, XM_006507768, XM_006507769, XM_006507767, XM_006507771, XM_006507770, XM_006507772, XM_006507773, XM_006507779, XM_006507784, XM_006507783, XM_006507781, XM_017322227, XM_006507782, XM_006507786, XM_006507778, XM_006507780, XM_006507785, XM_011241786, ENSMUST00000107196, ENSMUST00000098308, ENSMUST00000074273, ENSMUST00000107193, ENSMUST00000146563, MF276899, MF276900 | NP_035937, NP_001229975, XP_006507839, XP_006507840, XP_006507831, XP_006507832, XP_006507830, XP_006507834, XP_006507833, XP_006507835, XP_006507836, XP_006507842, XP_006507847, XP_006507846, XP_006507844, XP_017177716, XP_006507845, XP_006507849, XP_006507841, XP_006507843, XP_006507848, XP_011240088, ENSMUSP00000102814, ENSMUSP00000095910, ENSMUSP00000073885, ENSMUSP00000102811, Q91XM9, D3YWU0, E9Q2L2, D3YUZ8 | PDZ3 |
| 16 | 7:92126540-92126642 | 103 | NM_011807, NM_001243046, XM_006507776, XM_006507777, XM_006507768, XM_006507769, XM_006507767, XM_006507771, XM_006507770, XM_006507772, XM_006507773, XM_006507779, XM_006507784, XM_006507783, XM_006507781, XM_017322227, XM_006507782, XM_006507786, XM_006507778, XM_006507780, XM_006507785, XM_011241786, ENSMUST00000107196, ENSMUST00000098308, ENSMUST00000074273, ENSMUST00000107193, ENSMUST00000146563, MF276899, MF276900 | NP_035937, NP_001229975, XP_006507839, XP_006507840, XP_006507831, XP_006507832, XP_006507830, XP_006507834, XP_006507833, XP_006507835, XP_006507836, XP_006507842, XP_006507847, XP_006507846, XP_006507844, XP_017177716, XP_006507845, XP_006507849, XP_006507841, XP_006507843, XP_006507848, XP_011240088, ENSMUSP00000102814, ENSMUSP00000095910, ENSMUSP000000738858, ENSMUSP00000102811, Q91XM9, D3YWU0, E9Q2L2, D3YUZ | PDZ3 |
| η_1_ | 7:92180409-92181052 | 644 | XM_006507789 | non coding |  |
|  | 7:92180409-92181021 | 613 | XM_006507788 | non coding |  |
|  | 7:92180433-92181021 | 589 | XM_006507790 | non coding |  |
| η_2_ | 7:92234837-92235224 | 388 | XM_006507787 | non coding |  |
|  | 7:92234970-92235224 | 255 | NM_001243047 | non coding |  |
|  | 7:92235106-92235224 | 120 | AK039754 | Non coding |  |
| 17 | 7:92286491-92286605 | 115 | NM_011807, NM_001243046, NM_001243047, XM_006507776, XM_006507777, XM_006507768, XM_006507769, XM_006507767, XM_006507771, XM_006507770, XM_006507772, XM_006507773, XM_006507779, XM_006507784, XM_006507783, XM_006507781, XM_017322227, XM_006507782, XM_006507786, XM_006507778, XM_006507780, XM_006507785, XM_011241786, XM_006507789, XM_006507788, XM_006507790, XM_006507787, ENSMUST00000107196, ENSMUST00000098308, ENSMUST00000074273, ENSMUST00000107193, AK039754, MF276899, MF276900 | NP_035937, NP_001229975, NP_001229976, XP_006507839, XP_006507840, XP_006507831, XP_006507832, XP_006507830, XP_006507834, XP_006507833, XP_006507835, XP_006507836, XP_006507842, XP_006507847, XP_006507846, XP_006507844, XP_017177716, XP_006507845, XP_006507849, XP_006507841, XP_006507843, XP_006507848, XP_011240088, XP_006507852, XP_006507851, XP_006507853, XP_006507850, ENSMUSP00000102814, ENSMUSP00000095910, ENSMUSP00000073885, ENSMUSP00000102811, Q91XM9, D3YWU0, E9Q2L2, D3YUZ8 | Start codon (for NP_001229976, XP_006507852, XP_006507851, XP_006507853, XP_006507850)  SH3 |
|  | 7:92286491-92288935 | 2435 | ENSMUST00000146563 Retained intron |  | stop |
| 18 | 7:92375554-92375730 | 177 | NM_011807, NM_001243046, NM_001243047, XM_006507776, XM_006507777, XM_006507768, XM_006507769, XM_006507767, XM_006507771, XM_006507770, XM_006507772, XM_006507773, XM_006507779, XM_006507784, XM_006507783, XM_006507781, XM_017322227, XM_006507782, XM_006507786, XM_006507778, XM_006507780, XM_006507785, XM_011241786, XM_006507789, XM_006507788, XM_006507790, XM_006507787, ENSMUST00000107196, ENSMUST00000098308, ENSMUST00000074273, ENSMUST00000107193, AK039754, MF276899, MF276900 | NP_035937, NP_001229975, NP_001229976, XP_006507839, XP_006507840, XP_006507831, XP_006507832, XP_006507830, XP_006507834, XP_006507833, XP_006507835, XP_006507836, XP_006507842, XP_006507847, XP_006507846, XP_006507844, XP_017177716, XP_006507845, XP_006507849, XP_006507841, XP_006507843, XP_006507848, XP_011240088, XP_006507852, XP_006507851, XP_006507853, XP_006507850, ENSMUSP00000102814, ENSMUSP00000095910, ENSMUSP00000073885, ENSMUSP00000102811, Q91XM9, D3YWU0, E9Q2L2, D3YUZ8 | SH3 |
| 19 | 7:92386915-92386990 | 76 | NM_011807, NM_001243046, NM_001243047, XM_006507776, XM_006507777, XM_006507768, XM_006507769, XM_006507767, XM_006507771, XM_006507770, XM_006507772, XM_006507773, XM_006507779, XM_006507784, XM_006507783, XM_006507781, XM_017322227, XM_006507782, XM_006507786, XM_006507778, XM_006507780, XM_006507785, XM_011241786, XM_006507789, XM_006507788, XM_006507790, XM_006507787, ENSMUST00000107196, ENSMUST00000098308, ENSMUST00000074273, ENSMUST00000107193, AK039754, MF276899, MF276900 | NP_035937, NP_001229975, NP_001229976, XP_006507839, XP_006507840, XP_006507831, XP_006507832, XP_006507830, XP_006507834, XP_006507833, XP_006507835, XP_006507836, XP_006507842, XP_006507847, XP_006507846, XP_006507844, XP_017177716, XP_006507845, XP_006507849, XP_006507841, XP_006507843, XP_006507848, XP_011240088, XP_006507852, XP_006507851, XP_006507853, XP_006507850, ENSMUSP00000102814, ENSMUSP00000095910, ENSMUSP00000073885, ENSMUSP00000102811, Q91XM9, D3YWU0, E9Q2L2, D3YUZ8 | SH3 |
| 20 | 7:92417224-92417323 | 100 | NM_001243046, XM_006507776, XM_006507768, XM_006507769, XM_006507767, XM_006507771, XM_006507770, XM_006507779, XM_006507784, XM_006507783, XM_006507781, XM_006507782, XM_006507786, XM_006507778, XM_006507780, XM_006507785, XM_006507789, XM_006507788, XM_006507787, ENSMUST00000098308, ENSMUST00000074273, MF276899, MF276900 | NP_001229975, XP_006507839, XP_006507831, XP_006507832, XP_006507830, XP_006507834, XP_006507833, XP_006507842, XP_006507847, XP_006507846, XP_006507844, XP_006507845, XP_006507849, XP_006507841, XP_006507843, XP_006507848, XP_006507852, XP_006507851, XP_006507850, ENSMUSP00000095910, ENSMUSP00000073885, D3YWU0, E9Q2L2 |  |
| 21 | 7:92418088-92418133 | 46 | NM_011807, NM_001243047, XM_006507777, XM_006507772, XM_006507773, XM_017322227, XM_011241786, XM_006507790, ENSMUST00000107196, ENSMUST00000107193, AK039754 | NP_035937, NP_001229976, XP_006507840, XP_006507835, XP_006507836, XP_017177716, XP_011240088, XP_006507853, ENSMUSP00000102814, ENSMUSP00000102811, Q91XM9, D3YUZ8 |  |
| 22 | 7:92420632-92420673 | 42 | NM_001243046, XM_006507776, XM_006507768, XM_006507769, XM_006507767, XM_006507770, XM_006507772, XM_006507779, XM_006507784, XM_006507783, XM_006507781, XM_006507782, XM_006507786, XM_006507778, XM_006507785, XM_006507789, XM_006507788, XM_006507787, ENSMUST00000098308 | NP_001229975, XP_006507839, XP_006507831, XP_006507832, XP_006507830, XP_006507833, XP_006507835, XP_006507842, XP_006507847, XP_006507846, XP_006507844, XP_006507845, XP_006507849, XP_006507841, XP_006507848, XP_006507852, XP_006507851, XP_006507850, D3YWU0 |  |
| 23 | 7:92427691-92427741 | 51 | NM_011807, NM_001243046, NM_001243047, XM_006507776, XM_006507777, XM_006507768, XM_006507769, XM_006507767, XM_006507771, XM_006507770, XM_006507772, XM_006507773, XM_006507779, XM_006507784, XM_006507783, XM_006507781, XM_017322227, XM_006507782, XM_006507786, XM_006507778, XM_006507780, XM_006507785, XM_011241786, XM_006507789, XM_006507788, XM_006507790, XM_006507787, ENSMUST00000107196, ENSMUST00000098308, ENSMUST00000074273, ENSMUST00000107193, AK039754, MF276899, MF276900 | NP_035937, NP_001229975, NP_001229976, XP_006507839, XP_006507840, XP_006507831, XP_006507832, XP_006507830, XP_006507834, XP_006507833, XP_006507835, XP_006507836, XP_006507842, XP_006507847, XP_006507846, XP_006507844, XP_017177716, XP_006507845, XP_006507849, XP_006507841, XP_006507843, XP_006507848, XP_011240088, XP_006507852, XP_006507851, XP_006507853, XP_006507850, ENSMUSP00000102814, ENSMUSP00000095910, ENSMUSP00000073885, ENSMUSP00000102811, Q91XM9, D3YWU0, E9Q2L2, D3YUZ8 |  |
| 24 | 7:92428557-92428658 | 102 | NM_011807, NM_001243046, NM_001243047, XM_006507776, XM_006507777, XM_006507768, XM_006507769, XM_006507767, XM_006507771, XM_006507770, XM_006507772, XM_006507773, XM_006507779, XM_006507784, XM_006507783, XM_006507781, XM_017322227, XM_006507782, XM_006507786, XM_006507778, XM_006507780, XM_006507785, XM_011241786, XM_006507789, XM_006507788, XM_006507790, XM_006507787, ENSMUST00000107196, ENSMUST00000098308, ENSMUST00000074273, ENSMUST00000107193, AK039754, MF276899, MF276900 | NP_035937, NP_001229975, NP_001229976, XP_006507839, XP_006507840, XP_006507831, XP_006507832, XP_006507830, XP_006507834, XP_006507833, XP_006507835, XP_006507836, XP_006507842, XP_006507847, XP_006507846, XP_006507844, XP_017177716, XP_006507845, XP_006507849, XP_006507841, XP_006507843, XP_006507848, XP_011240088, XP_006507852, XP_006507851, XP_006507853, XP_006507850, ENSMUSP00000102814, ENSMUSP00000095910, ENSMUSP00000073885, ENSMUSP00000102811, Q91XM9, D3YWU0, E9Q2L2, D3YUZ8 | GUK |
| 25 | 7:92430997-92431169 | 173 | NM_011807, NM_001243046, NM_001243047, XM_006507776, XM_006507777, XM_006507768, XM_006507769, XM_006507767, XM_006507771, XM_006507770, XM_006507772, XM_006507773, XM_006507779, XM_006507784, XM_006507783, XM_006507781, XM_017322227, XM_006507782, XM_006507786, XM_006507778, XM_006507780, XM_006507785, XM_011241786, XM_006507789, XM_006507788, XM_006507790, XM_006507787, ENSMUST00000107196, ENSMUST00000098308, ENSMUST00000074273, ENSMUST00000107193, AK039754, MF276899, MF276900 | NP_035937, NP_001229975, NP_001229976, XP_006507839, XP_006507840, XP_006507831, XP_006507832, XP_006507830, XP_006507834, XP_006507833, XP_006507835, XP_006507836, XP_006507842, XP_006507847, XP_006507846, XP_006507844, XP_017177716, XP_006507845, XP_006507849, XP_006507841, XP_006507843, XP_006507848, XP_011240088, XP_006507852, XP_006507851, XP_006507853, XP_006507850, ENSMUSP00000102814, ENSMUSP00000095910, ENSMUSP00000073885, ENSMUSP00000102811, Q91XM9, D3YWU0, E9Q2L2, D3YUZ8 | GUK |
| 26 | 7:92437965-92438074 | 110 | NM_011807, NM_001243046, NM_001243047, XM_006507776, XM_006507777, XM_006507768, XM_006507769, XM_006507767, XM_006507771, XM_006507770, XM_006507772, XM_006507773, XM_006507779, XM_006507784, XM_006507783, XM_006507781, XM_017322227, XM_006507782, XM_006507786, XM_006507778, XM_006507780, XM_006507785, XM_011241786, XM_006507789, XM_006507788, XM_006507790, XM_006507787, ENSMUST00000107196, ENSMUST00000098308, ENSMUST00000074273, ENSMUST00000107193, AK039754, MF276899, MF276900 | NP_035937, NP_001229975, NP_001229976, XP_006507839, XP_006507840, XP_006507831, XP_006507832, XP_006507830, XP_006507834, XP_006507833, XP_006507835, XP_006507836, XP_006507842, XP_006507847, XP_006507846, XP_006507844, XP_017177716, XP_006507845, XP_006507849, XP_006507841, XP_006507843, XP_006507848, XP_011240088, XP_006507852, XP_006507851, XP_006507853, XP_006507850, ENSMUSP00000102814, ENSMUSP00000095910, ENSMUSP00000073885, ENSMUSP00000102811, Q91XM9, D3YWU0, E9Q2L2, D3YUZ8 | GUK |
| 27 | 7:92442604-92442695 | 92 | NM_011807, NM_001243046, NM_001243047, XM_006507776, XM_006507777, XM_006507768, XM_006507769, XM_006507767, XM_006507771, XM_006507770, XM_006507772, XM_006507773, XM_006507779, XM_006507784, XM_006507783, XM_006507781, XM_017322227, XM_006507782, XM_006507786, XM_006507778, XM_006507780, XM_006507785, XM_011241786, XM_006507789, XM_006507788, XM_006507790, XM_006507787, ENSMUST00000107196, ENSMUST00000098308, ENSMUST00000074273, ENSMUST00000107193, AK039754, MF276899, MF276900 | NP_035937, NP_001229975, NP_001229976, XP_006507839, XP_006507840, XP_006507831, XP_006507832, XP_006507830, XP_006507834, XP_006507833, XP_006507835, XP_006507836, XP_006507842, XP_006507847, XP_006507846, XP_006507844, XP_017177716, XP_006507845, XP_006507849, XP_006507841, XP_006507843, XP_006507848, XP_011240088, XP_006507852, XP_006507851, XP_006507853, XP_006507850, ENSMUSP00000102814, ENSMUSP00000095910, ENSMUSP00000073885, ENSMUSP00000102811, Q91XM9, D3YWU0, E9Q2L2, D3YUZ8 | GUK |
| 28 | 7:92444511-92447003 | 2493 | ENSMUST00000074273, ENSMUST00000107193 | ENSMUSP00000073885, ENSMUSP00000102811, E9Q2L2, D3YUZ8 | GUK,  Stop |
|  | 7:92444511-92446484 | 1974 | AK039754 |  |  |
|  | 7:92444511-92449106 | 4596 | XM_006507768, XM_006507769, XM_006507767, XM_006507771, XM_006507770, XM_006507772, XM_006507773, XM_006507779, XM_006507784, XM_006507783, XM_006507781, XM_017322227, XM_006507782, XM_006507786, XM_006507778, XM_006507780, XM_006507785, XM_011241786, XM_006507789, XM_006507788, XM_006507790, XM_006507787 | XP_006507831, XP_006507832, XP_006507830, XP_006507834, XP_006507833, XP_006507835, XP_006507836, XP_006507842, XP_006507847, XP_006507846, XP_006507844, XP_017177716, XP_006507845, XP_006507849, XP_006507841, XP_006507843, XP_006507848, XP_011240088, XP_006507852, XP_006507851, XP_006507853, XP_006507850 |  |
|  | 7:92444511-92449246 | 4736 | NM_011807, NM_001243046, NM_001243047, XM_006507776, XM_006507777, ENSMUST00000107196, ENSMUST00000098308, MF276899, MF276900 | NP_035937, NP_001229975, NP_001229976, XP_006507839, XP_006507840, ENSMUSP00000102814, ENSMUSP00000095910,Q91XM9, D3YWU0 |  |
