# Supplementary material for: Identification of a novel Dlg2 isoform differentially expressed in IFNβ-producing plasmacytoid dendritic cells
Source: BMC Genomics. 2018 Mar 12;19:194. doi: 10.1186/s12864-018-4573-5 (PMC6389146; doi:10.1186/s12864-018-4573-5)
Supplement: Supplementary file 4 — Dlg2 isoforms expressed in IFNβ-producing pDCs and cDCs. (PDF 560 kb) [file 12864_2018_4573_MOESM4_ESM.pdf]

Additional file 4

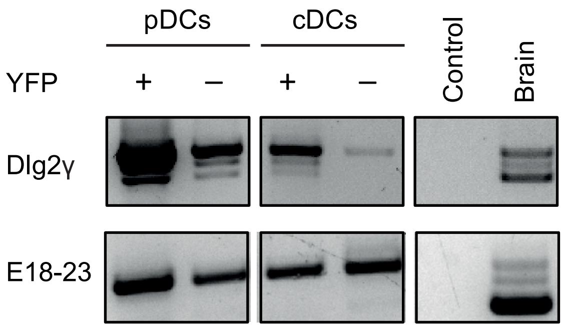

Additional file 4. *Dlg2* isoforms expressed in IFNβ-producing pDCs and cDCs. *Dlg2* Isoformes were analyzed by PCR on FACS sorted IFNβ/YFP^{+} and IFNβ/YFP^{-} pDCs and cDCs as well as brain tissue as described in the material and methods and figure legends of figure 2. Upper panel shows expression of the *Dlg2γ* isoform in IFNβ/YFP^{+} and IFNβ/YFP^{-} pDCs, cDCs and brain tissue. Lower panel shows the splicing pattern of the SH3-GK linker coding region.
